# The taxonomic composition of the donor intestinal microbiota is a major factor influencing the efficacy of faecal microbiota transplantation in therapy refractory ulcerative colitis

**DOI:** 10.1111/apt.14387

**Published:** 2017-10-20

**Authors:** P. Kump, P. Wurm, H. P. Gröchenig, H. Wenzl, W. Petritsch, B. Halwachs, M. Wagner, V. Stadlbauer, A. Eherer, K. M. Hoffmann, A. Deutschmann, G. Reicht, L. Reiter, P. Slawitsch, G. Gorkiewicz, C. Högenauer

**Affiliations:** ^1^ Division of Gastroenterology and Hepatology Department of Internal Medicine Medical University of Graz Graz Austria; ^2^ Theodor Escherich Laboratory for Medical Microbiome Research Medical University of Graz Graz Austria; ^3^ Institute of Pathology Medical University of Graz Graz Austria; ^4^ Barmherzige Brüder Hospital St. Veit an der Glan Austria; ^5^ BioTechMed‐Graz Interuniversity Cooperation Graz Austria; ^6^ Department of Pediatrics and Adolescent Medicine Medical University of Graz Graz Austria; ^7^ Barmherzige Brüder Hospital Graz Austria

## Abstract

**Background:**

Faecal microbiota transplantation is an experimental approach for the treatment of patients with ulcerative colitis. Although there is growing evidence that faecal microbiota transplantation is effective in this disease, factors affecting its response are unknown.

**Aims:**

To establish a faecal microbiota transplantation treatment protocol in ulcerative colitis patients, and to investigate which patient or donor factors are responsible for the treatment success.

**Methods:**

This is an open controlled trial of repeated faecal microbiota transplantation after antibiotic pre‐treatment (FMT‐group, n = 17) vs antibiotic pre‐treatment only (AB‐group, n = 10) in 27 therapy refractory ulcerative colitis patients over 90 days. Faecal samples of donors and patients were analysed by 16SrRNA gene‐based microbiota analysis.

**Results:**

In the FMT‐group, 10/17 (59%) of patients showed a response and 4/17 (24%) a remission to faecal microbiota transplantation. Response to faecal microbiota transplantation was mainly influenced by the taxonomic composition of the donor's microbiota. Stool of donors with a high bacterial richness (observed species remission 946 ± 93 vs no response 797 ± 181 at 15367 rps) and a high relative abundance of *Akkermansia muciniphila* (3.3 ± 3.1% vs 0.1 ± 0.2%), unclassified Ruminococcaceae (13.8 ± 5.0% vs 7.5 ± 3.7%), and *Ruminococcus* spp. (4.9 ± 3.5% vs 1.0 ± 0.7%) were more likely to induce remission. In contrast antibiotic treatment alone (AB‐group) was poorly tolerated, probably because of a sustained decrease of intestinal microbial richness.

**Conclusions:**

The taxonomic composition of the donor's intestinal microbiota is a major factor influencing the efficacy of faecal microbiota transplantation in ulcerative colitis patients. The design of specific microbial preparation might lead to new treatments for ulcerative colitis.

## 1 INTRODUCTION

Ulcerative colitis is an inflammatory bowel disease of the colon of unknown aetiology. One hypothesis of ulcerative colitis pathogenesis suggests that changes in the composition of colonic microbiota, called dysbiosis, cause activation of the mucosal immune system resulting in chronic inflammation.^1^, ^2^, ^3^, ^4^ Despite recent advances in the treatment of ulcerative colitis by drugs influencing different pathways of the immune system, still a considerable number of patients do not respond to medical therapy.^5^, ^6^

Faecal microbiota transplantation (FMT) is a therapeutic procedure aimed at restoring an altered intestinal microbiota by administrating faecal microorganisms from a healthy donor into the intestinal tract of a patient and thereby correcting dysbiosis. Faecal microbiota transplantation has been shown to be the most effective treatment of recurrent *Clostridium difficile* infection,^4^, ^7^, ^8^, ^9^, ^10^ a disease that is mainly triggered by an impaired intestinal colonisation resistance to pathogens due to depletion of the microbiota.^11^ As dysbiosis is believed to be a significant player in the pathogenesis of ulcerative colitis, the use of faecal microbiota transplantation has been studied in various case series^12^, ^13^, ^14^, ^15^, ^16^, ^17^, ^18^ and in four small randomised controlled trials.^19^, ^20^, ^21^, ^22^ The reported response rate to faecal microbiota transplantation in ulcerative colitis in published series varies between 0% and 100%.^12^, ^13^, ^14^, ^15^, ^16^, ^17^, ^18^, ^19^, ^20^, ^21^, ^22^ In a recent systematic review of studies, an average response rate of 49%‐55% and remission rate of 24%‐28% was reported.^17^ Three of the controlled trials^20^, ^21^, ^22^ showed a superiority of faecal microbiota transplantation compared to placebo while one trial failed^19^ to demonstrate a difference between faecal microbiota transplantation and the control group. So far, it remains unclear why some patients respond to this form of therapy while others do not. The large variability of faecal microbiota transplantation efficacy in ulcerative colitis patients raised several questions regarding the right faecal microbiota transplantation protocol or patient factors influencing treatment success.^17^

The aim of the current study was to establish a faecal microbiota transplantation treatment protocol with high clinical efficacy in therapy refractory ulcerative colitis patients and to investigate which patient or donor factors are responsible for the treatment success.

## 2 MATERIALS AND METHODS

### 2.1 Study design

This is an open prospective trial of repeated faecal microbiota transplantation after antibiotic pre‐treatment (FMT‐group) with a nonrandomised control group with antibiotic pre‐treatment only (AB‐group) in chronic active ulcerative colitis patients. The study was conducted at the Medical University of Graz, Division of Gastroenterology and Hepatology and in the convent hospital in St. Veit an der Glan in Austria from July 2012 to July 2014. The trial (DRKS00005331 on DRKS) was approved by the ethics committee of the Medical University Graz (EK23‐357ex10/11). Written informed consent was obtained from donors and patients, patients younger than 18 years required in addition a parent's consent.

All patients received antibiotic treatment including vancomycin 250 mg qid, paromomycin 250 mg tid and nystatin 10 mL (1 Million IE) qid for 10 days (antibiotic pre‐treatment). Subsequently, 5 faecal microbiota transplantation administrations were performed by endoscopy as described below in 14 days intervals in the FMT‐group (Figure S1). End of follow‐up was after 90 days. At each study visit, the total Mayo score, faecal calprotectin and a standard laboratory analysis were performed. The endoscopic Mayo score was initially assessed by two endoscopists and confirmed by an independent blinded endoscopist from electronic images of the site of most severe inflammation. Faecal samples for microbiota analyses were collected at each study visit (Figure S1).

Primary end point was the reduction of the total Mayo score on day 90.^23^ A reduction of the total Mayo score by ≥3 points was considered as a clinical response, whereas a drop of the Mayo score to ≤2 points was considered as remission. Patients with a response but no remission are denoted as partial responders. All clinical end point analyses were intention to treat (ITT). Patients who needed intensified therapy or terminated the study prematurely were assessed as treatment failures. Secondary end points were to find a specific microbial signature in responders vs nonresponders and between donors by 16S rRNA gene sequencing.

From June 2012 to July 2014, twenty‐seven patients were recruited. An interim analysis performed in October 2013 including 15 patients who had passed their primary end point at day 90, revealed a significant reduction in the total Mayo score during the antibiotic pre‐treatment without any faecal microbiota transplantation. From December 2013 to July 2014, 10 further recruited patients were treated as controls receiving only antibiotics without consecutive faecal microbiota transplantation (AB‐group), while 2 more patients of the FMT‐group finished follow‐up (FMT‐group, n = 17). The patients of the control group were tried to be matched according to disease activity and previously failed therapy for ulcerative colitis. Follow‐up in the AB‐group was clinically and endoscopically assessed on day 1, 14, and 90 including additional microbiota analysis, faecal calprotectin and laboratory analysis. Due to a poor short‐ and medium‐term tolerance of sole antibiotics in chronic active ulcerative colitis, further recruitment for this group was terminated (AB‐group, n = 10).

Statistical analysis of clinical data was performed using spss version 15.0 (spss, Chicago, Illinois, USA). Categorical data were compared by Fisher's exact test, or the chi‐square test, as appropriate; Student's *t* test was used for comparison of continuous variables, and the Mann‐Whitney U‐test if data were not normally distributed. *P*‐values <.05 were considered statistically significant.

#### 2.1.1 Study population‐patients

Eligible patients with chronic active ulcerative colitis were aged between 16 and 80 years. Diagnosis was based on the current guidelines of the European Crohn`s and Colitis Organisation.^24^ Active ulcerative colitis was defined as a total Mayo score ≥4 and an endoscopic subscore ≥1. All patients had treatment failures for at least one immunosuppressive agent and/or anti‐TNF‐antibody. Antibiotics and faecal microbiota transplantation were given as an add‐on treatment to concomitant therapy provided that they were on a stable dose (no change in the dose of 5‐ASA, immunosuppressant and anti‐TNF therapy for 8 weeks, and a stable corticosteroid dose for at least two weeks) and still presenting with active disease. All concomitant therapies had to be continued at similar doses during the study. A tapering of steroids was allowed. Prior to antibiotic pre‐treatment coexisting infections with *C. difficile* or any other enteric pathogens as *Salmonella, Shigella, Campylobacter, Yersinia* and EHEC were excluded by culture and PCR (*C.difficile* toxin B gene). Not eligible for the study were ulcerative colitis patients with any blood clotting disorder or treatments with oral anticoagulants, known allergies to the provided antibiotics or women who were pregnant.

#### 2.1.2 Study population‐donors

Donors were related or unrelated volunteers, had to be ≥18 years and were selected according to the Austrian guidelines for screening stool donors for faecal microbiota transplantation.^25^ Donors had to ensure that they had no antibiotic therapy or enteric infections within 3 month before stool donation. Eligible donors were allowed to donate faeces for more than one patient but the same donor had to serve one patient throughout the 5 repeated faecal microbiota transplantations. Donor screening was repeated every 6 month or sooner in the case of certain risks for infectious diseases (eg, vacation in countries with a high risk for gastrointestinal infections).

Fourteen donors were eligible for the study. Two donors (donor no. 1 and 3) donated for more than one patient (donor no. 1 for A,B and C; donor no. 3 for E and K). The majority of the donors were unrelated, anonymous volunteers (n = 6) or friends (n = 3), 2 were partners and 3 were relatives. The mean age of the donor group was 38 years (range 27‐54) and 62% were male.

#### 2.1.3 Donor stool preparation and protocol for faecal microbiota transplantation

For each faecal microbiota transplantation, fresh donor stool was collected in special vessels (2500 mL vessels for disposable specimen; LP Italiana Spa, Milano, Italy) and stored at 4°C. Donor stool preparation was performed under biohazard level 2 conditions according to the Austrian faecal microbiota transplantation guideline.^25^ Within 6 hours after donation, a minimum of 50 g of stool was diluted with sterile saline (200‐500 mL), homogenised and filtered in a one‐step procedure using a standard household blender with integrated metal sieve. (Phillips HR 2084/90 Essence). A total of 250 to 500 mL faecal suspension was placed into syringes for immediate application. An aliquot of the original donor stool was collected for microbiota analysis. Antibiotic pre‐treatment was stopped 36 hours prior to the first faecal microbiota transplantation.

The day following bowel preparation patients of the FMT‐group underwent total ileocolonoscopy. Overall, 250 to 500 mL of the faecal suspension was applied into the terminal ileum and the right colon after standard bowel preparation using a PEG‐based solution (Moviprep^®^, Norgine. Amsterdam, Netherlands). At 14 days intervals, 4 more repeated faecal microbiota transplantations (day 14, day 28, day 42 and day 56) were performed by flexible sigmoidoscopy without any bowel preparation by deposing a freshly prepared faecal suspension (250‐500 mL) of the same donor into the left colon.

### 2.2 DNA extraction, 16S rRNA gene amplification and sequencing

Stool samples of patients and donors were immediately frozen and stored at −20°C. DNA extraction from stool samples was performed by mechanical lysis with a MagnaLyser Instrument (Roche Diagnostics, Mannheim, Germany) and subsequent total bacterial genomic DNA isolation with the MagNA Pure LC DNA Isolation Kit III (bacteria, fungi) in a MagNA Pure LC 2.0 Instrument (Roche Diagnostics) according to the manufacturer's instructions.^12^ For amplification of bacterial 16S rRNA gene, the template‐specific sequence 515F‐5′‐GTGCCAGCMGCCGCGGTAA‐3′ and 806R‐5′‐GGACTACHVGGGTWTCTAAT‐3′, targeting the hypervariable region V4 of the 16S rRNA gene were used.^26^ PCR reactions for each sample were performed in triplicates. Subsequently, the amplicons were purified according to standard procedures, quantified, pooled and sequenced with the MiSeq Reagent Kits v3 (600 cycles, Illumina, Eindhoven, Netherlands) according to manufacturer's instructions with 20% OhiX (Illumina). The generated FASTQ files were used for microbiota analysis.

### 2.3 Microbiota analysis

Raw files from Illumina MiSeq were processed according to the standard MiSeq SOP of mothur.^27^, ^28^ Sequencing errors were reduced using mothur's pre.cluster command to remove sequences that arose due to sequencing errors. Chimeras were removed with UCHIME^29^ and nonbacterial contaminants as chloroplasts and mitochondria were removed by classify.seqs and remove.lineage using the ribosomal database project (RDP) training set (v.14). The high quality reads were aligned to the SILVA database (v.119).^30^ For an operational taxonomic unit (OTU)‐based analysis of the dataset; the processed fasta files from mothur were then introduced into QIIME version 1.8.0.^31^ Open‐reference OTU picking strategy was performed according to OTU similarity clustering with UCLUST^32^ on a similarity score of 97% and Greengenes reference 13.08 was used.^33^ Subsequently, diversity analyses were performed in QIIME according to the core_diversity_analysis.py workflow. For the analyses, samples were rarefied to at least 15376 sequences/sample. For statistical comparisons of alpha‐diversity metrics (observed species, chao1, PD whole tree and Simpson's index), a nonparametric *t* test with 999 Monte‐Carlo permutations was performed and the Bonferroni method was used for multiple comparison corrections. Calculated beta diversity metrics (Bray Curtis, unweighted and weighted UniFrac) were compared by using the nonparametric ANOSIM measure. Significant differences in relative abundances of taxa were calculated by using a nonparametric Kruskal‐Wallis test using false discovery rate (FDR) correction and linear discriminant analysis effect size (LEfSe).^34^ *P*‐values below 0.05 were considered statistically significant (*P <.05; **P <.01; ***P <.001). Presented values are always mean ± SEM if not indicated otherwise. Only samples of patients treated per protocol were included in the analysis.

## 3 RESULTS

### 3.1 Clinical outcomes

A total of 27 patients were included in the study. Twenty‐five patients (17/17 in the FMT‐group and 8/10 in the AB‐group) finished the study (Figure S2). Baseline patient characteristics are shown in Table 1. There was no statistical significant difference between groups except for sex.

**Table 1:** Baseline characteristics of patients

	FMT‐group (n = 17)	AB‐group (n = 10)	*P* value^a^
Mean age, y ± SD	44 ± 18	36 ± 13	.20
Male sex, n (%)	14 (82)	3 (30)	.013
Mean disease duration, y ± SD	8 ± 8	7 ± 6	.53
Extent of disease, n (%)			
E1, proctitis	1 (6)	1 (10)	.68
E2, left sided	10 (59)	7 (70)	
E3, pancolitis	6 (35)	2 (20)	
Concomitant drug treatment, n (%)	14 (82)	10 (100)	.16
Mesalazine oral	9 (53)	8 (80)	.21
Immunosuppressants	5 (29)	6 (60)	.12
Anti‐TNF	1 (6)	2 (29)	.44
Systemic corticosteroids	10 (59)	3 (30)	.063
Prior immunosuppressants, n (%)	13 (76)	7 (70)	.67
Prior anti‐TNF, n (%)	10 (59)	4 (40)	.29
Mean total Mayo score at inclusion ± SD	8.9 ± 1.6	8.1 ± 3.1	.54
Endoscopic Mayo subscore at inclusion, n (%)			
Mayo 1	1 (6)	1 (10)	.55
Mayo 2	5 (29)	3 (30)	
Mayo 3	11 (65)	6 (60)	
Disease severity by the total Mayo score at inclusion n (%)			
Mild (3‐5 points)	1 (6)	2 (20)	.33
Moderate (6‐10 points)	13 (76)	6 (60)	
Severe (11‐12 points)	3 (18)	2 (20)	

AB, antibiotics; FMT, faecal microbiota transplantation; SD, standard deviation; y, year; Anti‐TNF, antitumour necrosis factor alpha antibodies.

a Fisher's exact test or chi‐square test (as appropriate), unpaired *t* test for age, Mann‐Whitney U‐test for disease duration and for total Mayo score at inclusion.

Repeatedly performed faecal microbiota transplantation after antibiotic pre‐treatment (FMT‐group) resulted in a clinical response in 10 out of 17 (59%) patients on day 90 (Table S1, Figure S3). Of the responders, 4 patients (24%) achieved clinical remission and 6 (35%) a partial response (Table S1), all patients with remission had also an endoscopic Mayo score of 0 or 1 indicative of mucosal healing. In the AB‐group, only 1 of 10 (10%) patients classified as partial responder at day 90 (Figure S3). In the FMT‐group, 10/17 patients had steroid dependent active disease and were treated with faecal microbiota transplantation as an add‐on therapy. The average amount of steroids in these patients at baseline was 23.1 mg/d and significantly dropped at day 90 to 3.5 mg/d (*P* <.05). Of the 10 patients on steroids at baseline, steroids were discontinued in 4 patients (2 remission, 1 partial response, 1 no response) while 6 patients were still on steroids at day 90, (3 partial response, 3 no response). In a univariate analysis factors predicting treatment response were male sex, a higher total Mayo score at baseline and if patients received faecal microbiota transplantation (Table S2). Table S1 shows the clinical characteristics and Table S3 the laboratory parameters of patients of the FMT‐group at baseline and after antibiotic therapy according to their response to faecal microbiota transplantation.

Long‐term follow‐up data of patients receiving FMT were available in 14 of 17 patients (mean 39 months, range 18 to 50). Of the 4 patients in remission after FMT, none required additional immunosuppressive or biologic therapy and are either in remission or have mild disease activity. However, all other 10 patients with long‐term follow‐up required either a new immunosuppressive and/or biologic therapy. Two patients, one partial responder and 1 nonresponder underwent colectomy.

Donor no. 1 served 3 patients, all of them were nonresponders, donor no. 3 served 2 patients, one showed clinical remission (reduction of total Mayo score from 8 to 2 points), the other a partial response (reduction in total mayo score from 8 to 5) at day 90, however, the second patient achieved clinical remission another 4 weeks later (day 120).

Antibiotic pre‐treatment by itself led to a decrease in the total Mayo score by a mean of 2 points for all 27 patients (FMT‐ and AB‐group) after 10 days, with 5 of 27 patients fulfilling the criteria of a response. However if antibiotics were not followed by faecal microbiota transplantation (AB‐group), 5/10 patients could not finish the study according to the protocol since they required additional therapy due to *C. difficile* infection after day 14 (n = 3), antibiotic‐associated diarrhoea (*C. difficile* negative, n = 1) and aggravation of ulcerative colitis (n = 1). In contrast, only 1/17 patient of the FMT‐group needed additional therapy (low‐dose steroids) for worsening of ulcerative colitis which he started by himself after day 3 (Figure S2).

### 3.2 Microbiota analysis

#### 3.2.1 Differences in the microbiota of patients and donors according to treatment response

**Figure 1:**
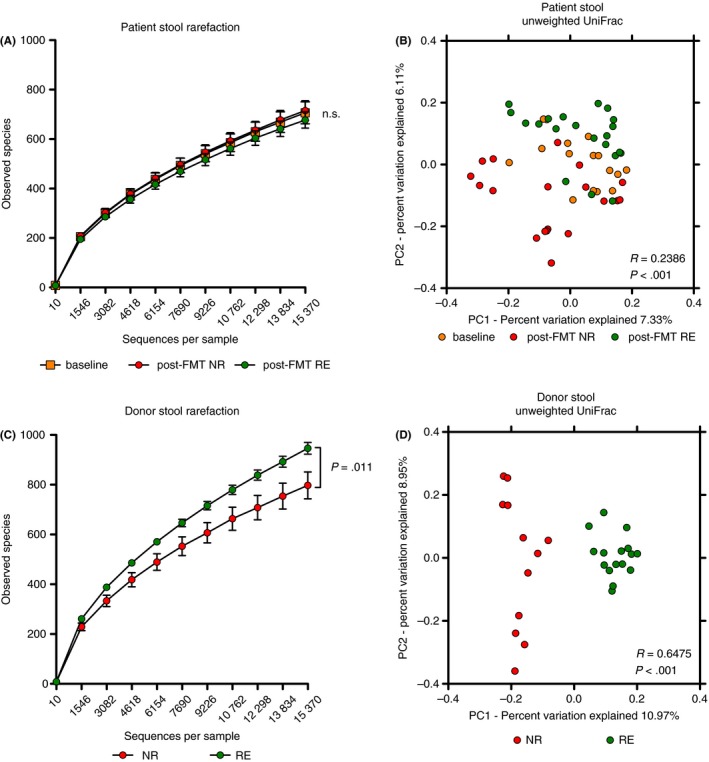
Microbial composition of the donor stools determines efficacy of faecal microbiota transplantation in chronic active ulcerative colitis. A, No significant difference in microbial richness (observed species) of stool samples from recipients pre‐ and post‐faecal microbiota transplantation (FMT) when stratified according to treatment response (orange: baseline at day −10, n = 16; red: post‐FMT no response (NR), n = 19; green: post‐FMT remission (RE), n = 21). B, Principle coordinates analysis (PCoA) of unweighted UniFrac distance shows a significant clustering of patient stools comparing baseline samples (at day −10; orange) and post‐FMT (red: NR; green: RE). The low R‐value = 0.2386 indicates an overall weak grouping. C, Donor stools leading to remission in recipients showed a significantly increased richness compared to donor stools leading to no response (red: NR, n = 12; green: RE, n = 16; *P* = .011). Plots show mean ±  SEM,* P*‐values given for difference at 15376 sequences per sample based on two‐sample *t* test. D, PCoA indicated significantly different microbial community types with a strong grouping when donor stools were stratified according to response in the recipient (red: NR, n = 12; green: RE, n = 16; *P*<.001, R = 0.6475, ANOSIM)

Comparative microbial community profiling was performed with faecal specimens to ascertain potential microbial signatures associated with efficacy or failure of repeated faecal microbiota transplantations. The taxonomic composition of the patient and donor faecal microbiota is shown in Figure S4. Comparing the recipients’ stool specimens before and after faecal microbiota transplantation, the latter stratified according to remission or no response, there was no statistically significant differences in richness (Figure 1A). PCoA analysis (measure: unweighted UniFrac distance) of the recipients’ stools indicated a significantly different microbial community structure comparing pre‐faecal microbiota transplantation and post‐faecal microbiota transplantation samples, although stratification of groups was only weak (Figure 1B, *P*‐value <.001, R‐value of 0.2386; ANOSIM). In contrast, when donor stools were analysed and stratified according to treatment response in the recipient, a strong separation of groups was evident. Donor microbiotas associated with remission showed a significantly higher bacterial richness and diversity compared to donors associated with no response. (Figure 1C, Figure S5), this was not evident when comparing donor microbiotas leading to response and donors leading to no response (Figure S6). PCoA indicated a significantly different microbial community structure with a strong separation of groups (Figure 1D, measure: unweighted UniFrac; R‐value 0.6475, *P*<.001; ANOSIM and Figure S7). The same separation of groups is seen if only one sample per donor was included for analysis (FigureS7B). Although also significant, the separation was less pronounced when analysed for donor microbiotas leading to response and no response (Figures S8 & S9).

**Figure 2:**
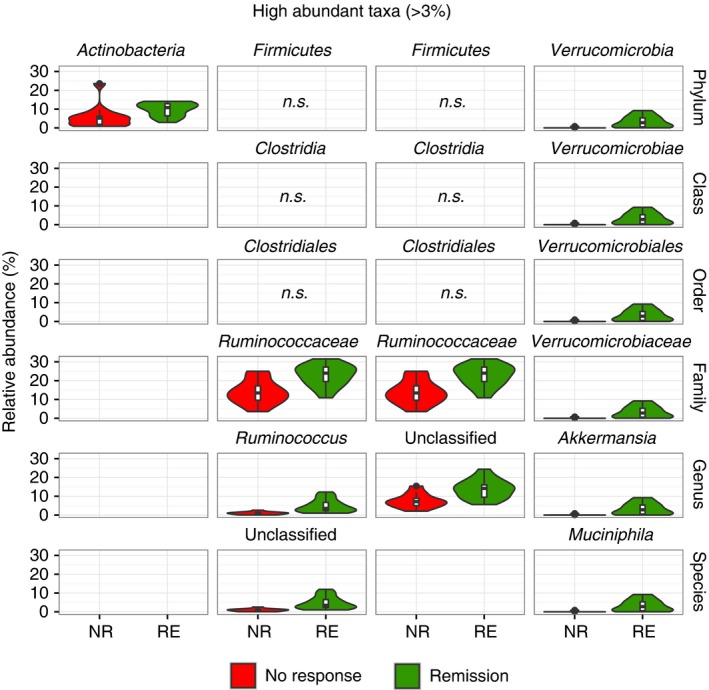
Taxonomic signature in donor stools associated with treatment response. Significantly different taxa from phylum to species level in the donors’ microbiota associated to NR and RE in the recipient according to high abundant taxa (mean >3%). Plotted taxa shown are significantly different between both treatment response groups based on a nonparametric Kruskal‐Wallis test with FDR correction in QIIME (*P *<.05) as well as on discriminatory features calculated by LEfSe (*P *<.05 and LDA‐score >2; red: no response, NR, n = 12; green: remission, RE, n = 16)

**Figure 3:**
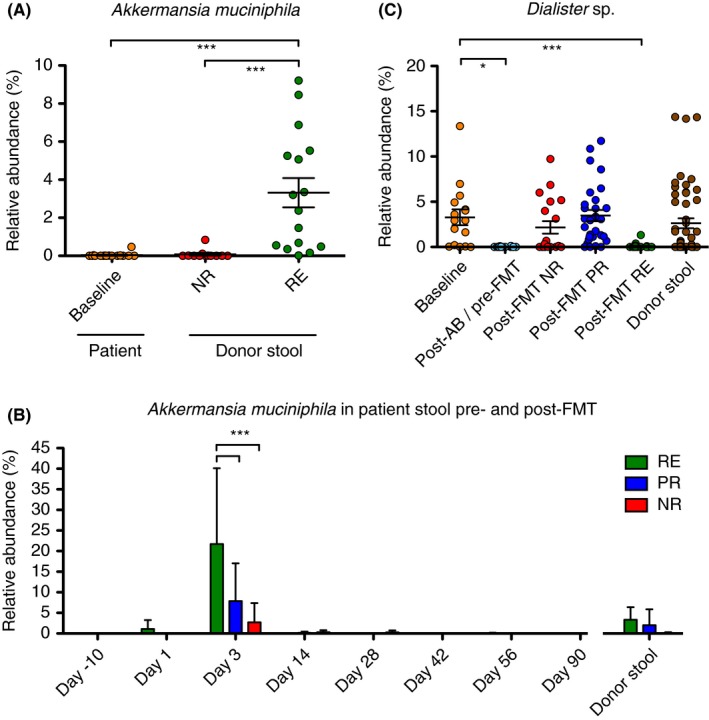
Efficacy of faecal microbiota transplantation correlates to abundance of specific taxa in donors and patients after faecal microbiota transplantation. A, Relative abundance of *A. muciniphila* in patient stools pre‐faecal microbiota transplantation(FMT) (orange) and donor stools according to treatment success of faecal microbiota transplantation (red: no response, NR; green: remission, RE; Kruskal‐Wallis test, Dunn's multiple comparison test, ***P <.001). B, A high relative abundance of *A. muciniphila* was detected in patients responding to faecal microbiota transplantation on the first day after the initial faecal microbiota transplantation application (day 3), with a significant increase in RE patients compared to partial response (PR) or NR. In contrast, *A. muciniphila* abundance was low or nondetectable before faecal microbiota transplantation (day‐10, day 1). No long‐term colonisation of *A. muciniphila* was seen in the other post‐faecal microbiota transplantation samples (day 14, 28, 42, 56 and 90) from ulcerative colitis patients (red: NR; blue: PR; green: RE; n = 2‐7, Two‐way ANOVA, Bonferroni post‐test, ***P <.001). C, Relative abundance of *Dialister* sp. in stools of ulcerative colitis patient at baseline (orange, n = 16), post‐antibiotic treatment (light‐ blue, n = 22) and post‐FMT according to treatment response (d3 to d90; red: NR, n = 19; blue: PR, n = 29; green: RE, n = 21) and in donor stools (brown, n = 51, Kruskal‐Wallis test, Dunn's multiple comparison test, *P <.05, ***P <.001)

**Figure 4:**
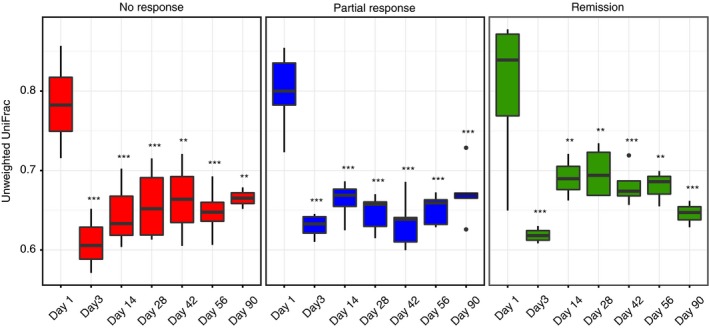
Engraftment of the donor microbiota is unrelated to treatment response by repeated faecal microbiota transplantations. A significant and continuous decline of UniFrac distance in all response groups when donor stools were compared to recipients indicates engraftment in all groups. A lower distance indicates a higher similarity of the microbial community composition comparing donors and recipients (red: no response, NR; blue: partial response, PR; green: remission, RE; n = 2‐5; Two‐way ANOVA, Bonferroni post‐test **P <.01, ***P <.001)

We next sought to identify the taxa causing these differences. Since the signal related to efficacy in faecal microbiota transplantation was strongest in donor samples, we first compared the donor microbiotas, again stratified according to response in the recipient. Donor stools associated with remission showed a significantly higher relative abundance of Actinobacteria, unclassified Ruminococcaceae, an unclassified *Ruminococcus* sp. and *Akkermansia muciniphila* besides certain other taxa with low abundance (Figure 2, Figures S10 & S11), the latter two were also significantly different using a more stringent Bonferroni correction (data not shown). Also when analysing only one sample per donor, *A. muciniphila* and *Ruminococcus* spp. are still significantly increased in donor samples leading to remission post‐faecal microbiota transplantation (Figure S12). Unclassified *Ruminococcus* sp. and *Akkermansia muciniphila* were also increased, although less significantly, when comparing donors leading to response vs donors leading to no response (Figure S13). *A. muciniphila* was nearly absent in baseline samples of patients (ie, active ulcerative colitis) as well as in donor stools leading to no response (Figure 3A). In recipients, *A. muciniphila* was significantly increased the day after the initial faecal microbiota transplantation application (day 3) in patients achieving remission but not in patients showing a partial‐ or no response, as well as in later time‐points (Figure 3B). Conversely, *Dialister* sp. was significantly reduced in patients achieving remission after faecal microbiota transplantation in addition to samples after antibiotic treatment (day 1) (Figure 3C). Interestingly, engraftment of the donor microbiota per se seemed not be a factor for treatment success, since phylogenetic distance (unweighted UniFrac) clearly indicated that all recipients’ microbiotas, regardless of response, shifted towards the respective donor microbiota (Figure 4).

#### 3.2.2 Effect of antibiotic pre‐treatment on the faecal microbiota in ulcerative colitis patients

Antibiotic pre‐treatment for 10 days resulted in a significantly decreased species richness (Figure S14A). Faecal microbiota transplantation restored species richness while an increase in richness after the antibiotic treatment was not evident in patients without faecal microbiota transplantation (AB‐group, Figure S14B). The latter finding could explain the high dropout rates in the AB‐control group due to *C. difficile* infections and diarrhoea, since a decreased microbial richness is a risk factor for this infection due to an impaired colonisation resistance.

A subgroup of 5 patients (from the FMT‐ and AB‐group) achieved a clinical response already after the 10 days antibiotic pre‐treatment, but none achieved remission. These patients showed significant differences in microbiota composition at the taxonomic level compared to patients showing no response after antibiotic treatment. An inverse relation in the relative abundances of Streptococcaceae and Enterobacteriaceae, wherein partial response was associated with decreased Enterobacteriaceae loads and increased Streptococcaceae loads were noted (Figure S15).

## 4 DISCUSSION

The results of this study showed that the composition of the donor microbiota seems to be one crucial factor for response to faecal microbiota transplantation in patients with therapy refractory chronic active ulcerative colitis. The faecal microbiota of stool donors used in patients with the best treatment success to faecal microbiota transplantation were characterised by a different taxonomic composition as a significant higher abundance of *A. muciniphila* and had a higher bacterial richness compared to a donor microbiota of unsuccessfully treated patients.

A specific donor microbiota factor for successful faecal microbiota transplantation in ulcerative colitis was also supported by Moayyedi et al.^20^ In their study, ulcerative colitis patients treated with the stool of one specific donor showed a higher response rate to faecal microbiota transplantation than patients who received the stool from other donors but a specific microbiota signature, associated with treatment response, was not reported. Also in line with our results is a small case series of inflammatory bowel disease patients treated by faecal microbiota transplantation, wherein a higher bacterial richness of donors was associated with faecal microbiota transplantation response in patients.^35^ However, the findings of the latter study need to be interpreted with caution since treatment response was not exactly defined, both Crohn's disease and ulcerative colitis patients were included in the analysis and different faecal microbiota transplantation application protocols were used.^35^ Specific selection criteria for donors to achieve better faecal microbiota transplantation results in inflammatory bowel disease have thereafter been called for,^36^ but specific criteria for selection are still lacking.

We could identify certain bacterial taxa in the donor microbiota, which were associated with treatment response to faecal microbiota transplantation, mainly *A. muciniphila,* and certain Ruminococcaceae. We postulate that the taxonomic composition of the donor microbiota with the presence or absence of specific taxa determines in part the treatment response to faecal microbiota transplantation in ulcerative colitis. Especially, a high abundance of *A. muciniphila* in the donor microbiota seems favourable for the treatment response of faecal microbiota transplantation. It has been shown that *A. muciniphila* loads are significantly decreased in inflammatory bowel disease patients,^37^ and *A. muciniphila* is important for intestinal wound‐healing in a mouse model of intestinal injury.^38^ The fact that we could not observe long‐term colonisation with *A. muciniphila* in patients responding to faecal microbiota transplantation, despite high levels in the applied donor stools, needs to be clarified. The finding that *A. muciniphila* is able to mediate its anti‐inflammatory properties also indirectly via mediators like outer‐membrane vesicles (OMVs)^39^ is a possibility. Alternatively, *A. muciniphila* could be just an indicator of a beneficial intestinal microbiota with anti‐inflammatory properties in ulcerative colitis patients, rather than acting by itself, as *A. muciniphila* has also been shown to promote inflammation in an animal colitis model.^40^ Other taxa in the donor microbiota associated with response were Ruminococcaceae including the genus *Ruminococcus*. Noteworthy, these taxa did not represent *Faecalibacterium prausnitzii*, also a member of Ruminococcaceae, but matched to *Ruminococcus bromii* and *Ruminococcus champanellensis* (Figure S16). A possible beneficial property of these Ruminococcaceae is their ability to process nondigestible carbohydrates thereby promoting the production of short chain fatty acids, which are known to be decreased in inflammatory bowel disease.^41^ In the study by Vermeire et al, Ruminococcaceae were claimed to be transferred from the donor to two of the patients responding to faecal microbiota transplantation.^35^ For clarification, the taxa *Ruminococcus gnavus* and *Ruminococcus torques*, which have been demonstrated to be increased in ulcerative colitis patients^37^ and have also to be associated with donors leading to no response to faecal microbiota transplantation in ulcerative colitis^42^ are not the taxa found in our analysis. We have no explanation why *F. prausnitzii*, which is reported to be depleted in inflammatory bowel diseases and has anti‐inflammatory properties^43^, ^44^ was not associated to a beneficial donor microbiota. Maybe other Ruminococcaceae found in these donors share similar anti‐inflammatory properties like *F. prausnitzii* another possible explanation is that viable *F. prausnitzii,* which is oxygen sensitive might have been inactivated due to the mixing procedure under aerobic conditions. The importance of the donor microbiota might also explain the large variations in response rates of faecal microbiota transplantation in ulcerative colitis reported in previous studies ranging between 0% and 100%^12^, ^13^, ^14^, ^15^, ^16^, ^17^, ^18^, ^19^, ^20^, ^21^, ^22^ and also why one randomised study showed no significant effect of FMT.^19^ The concept of mixing stool from different donors, as performed by Paramsothy et al might increase the likelihood of transferring a beneficial microbiota to ulcerative colitis patients and could therefore explain the superiority of faecal microbiota transplantation over placebo in this study.^21^ As the abundance of the taxa found to be associated with treatment response in our study is influenced by diet, it is important for future studies to also assess the diet of the donors which might be a contributing factor for response to FMT.

In contrast to the signature in the donor microbiota, there was no clearly discernible pattern in the microbiota of recipients showing a faecal microbiota transplantation response. The only phylotype differing significantly between groups was *Dialister* sp., which was lowest in patients with remission after faecal microbiota transplantation. Increased loads of *Dialister* sp. were reported in inflammatory bowel disease and also spondyloarthritis.^45^, ^46^ Since we also observed similar *Dialister* levels in healthy donors and in patients with active ulcerative colitis, the relevance of this finding remains unclear, besides indicating response to faecal microbiota transplantation. In contrast to the results of previous studies, we neither observed a significant difference in microbial diversity between remission cases and nonresponders^19^, ^21^ nor a difference in efficacy of engraftment of the transplant between response groups^19^, ^42^ (Figure 4). The lack of increase in microbial richness in our trial might be explained by the preceding antibiotic therapy which was not used in the previous randomised controlled trials.

Other factors like a heterogeneous patient population or the faecal microbiota transplantation protocol might also influence the efficacy of faecal microbiota transplantation. In contrast, to Moayyedi et al^20^ describing a trend towards a better response in patients with ongoing immunosuppressive therapy and an overall shorter disease duration, we identified male sex and a higher total Mayo score as predicting factors of response (Table S2). Regarding to factors of the faecal microbiota transplantation protocol, we used repeated faecal microbiota transplantation application in the lower GI tract with antibiotic pre‐treatment. Antibiotic pre‐treatment was chosen to decrease intestinal colonisation resistance since in a previous study, we observed a large variation in engraftment of the donor microbiota after faecal microbiota transplantation in ulcerative colitis.^12^ The antibiotics were chosen because they are nonabsorbable and have a broad spectrum against Gram‐positive and Gram‐negative bacteria, thereby possibly leading to a major change in the intestinal microbiota. The antimycotic was added to avoid candida overgrowth by antibiotics.^47^ In this context, the changes of the patients’ microbiota in ulcerative colitis patients showing a response already after 10 days of antibiotic pre‐treatment deserve attention. Partial responders to antibiotics showed significantly decreased levels of Enterobacteriaceae, which commonly include pro‐inflammatory pathobionts,^48^ but high levels of certain Streptococcaceae*,* including the species *S. thermophilus* (Figure S15), which might be beneficial in this context. *S. thermophilus* and other members of lactic acid bacteria are used as probiotics with positive effects in pouchitis.^49^ These findings might help to understand the varying effects of antibiotics in ulcerative colitis.^50^

Our protocol of repeated faecal microbiota transplantation after antibiotic pre‐treatment was well tolerated and resulted in clinical improvement in the majority and to remission in one‐fourth of patients with therapy refractory ulcerative colitis. This is in line with the results of the randomised controlled trials, however the patient population studied here is particularly difficult to treat since all patients failed either previous immunosuppressive or biologic therapy or even failed to both therapies. On the other hand, antibiotic treatment without faecal microbiota transplantation was poorly tolerated in patients and resulted only in short‐term improvement of disease activity. Of our patients treated with antibiotics, 50% alone experienced significant side‐effects like *C. difficile* infections, antibiotic‐associated diarrhoea and exacerbation of ulcerative colitis. Since microbial richness was decreased in the long‐term by antibiotics alone (Figure S14), we assume that these side‐effects were caused by a loss of intestinal colonisation resistance leading to infections with intestinal pathogens as *C. difficile*.

The limitations of our study are the small numbers of patients and that patients were not randomised to the treatments arms. Since the antibiotic control group was stopped because of poor tolerability, there were differences in the number of patients in both groups, furthermore several patients in the antibiotic control group were nonadherence to the treatment protocol. In the FMT‐group, a larger proportion of patients, although nonstatistically different to the control group were on steroids at baseline. The comparison between the FMT‐group and the AB‐group needs therefore to be interpreted with caution. It is therefore possible that additional factors not appearing significant in this study as concomitant therapies of ulcerative colitis patients, microbiota composition of patients as well as other taxa in the donor microbiota might also influence efficacy of faecal microbiota transplantation. Despite these limitations, we observed a strong signal regarding the composition of the donor microbiota in relation to treatment response to faecal microbiota transplantation even if only one sample per donor was analysed, suggesting a biologic relevant finding, although it needs to be mentioned that because of the small numbers of donors false‐positive results due to multiple testing are still possible.

This study provides further evidence that repeated faecal microbiota transplantation is effective in treating patients with therapy refractory ulcerative colitis. It suggests an important effect of the donor microbiota to the treatment success. These findings might lead to a more specific donor selection for faecal microbiota transplantation and to the development of specific microbial preparations for the treatment of ulcerative colitis in the future.

## AVAILABILITY OF DATA AND MATERIAL

The datasets generated and analysed during the current study are available in the European nucleotide archive (ENA) repository under the Primary accession number PRJEB11841 (Secondary accession ERP013257).

## AUTHORSHIP

*Guarantor of the article*: Christoph Högenauer.

*Authors’ contributions*: PK, CH: design of study; PK, PW, GG, CH: drafting of manuscript; PK, HPG, WP, AE, MW, VS, KMH, AD, GR, LR, PS, CH: acquisition of data; HW, BH, PW statistical analysis; PW, BH, GG: microbiota analysis. All authors read and approved the final manuscript.
