# Supplementary material for: The taxonomic composition of the donor intestinal microbiota is a major factor influencing the efficacy of faecal microbiota transplantation in therapy refractory ulcerative colitis
Source: Aliment Pharmacol Ther. 2017 Oct 20;47(1):67–77. doi: 10.1111/apt.14387 (PMC5765501; doi:10.1111/apt.14387)
Supplement: Supplementary file 1 [file APT-47-67-s001.pdf]

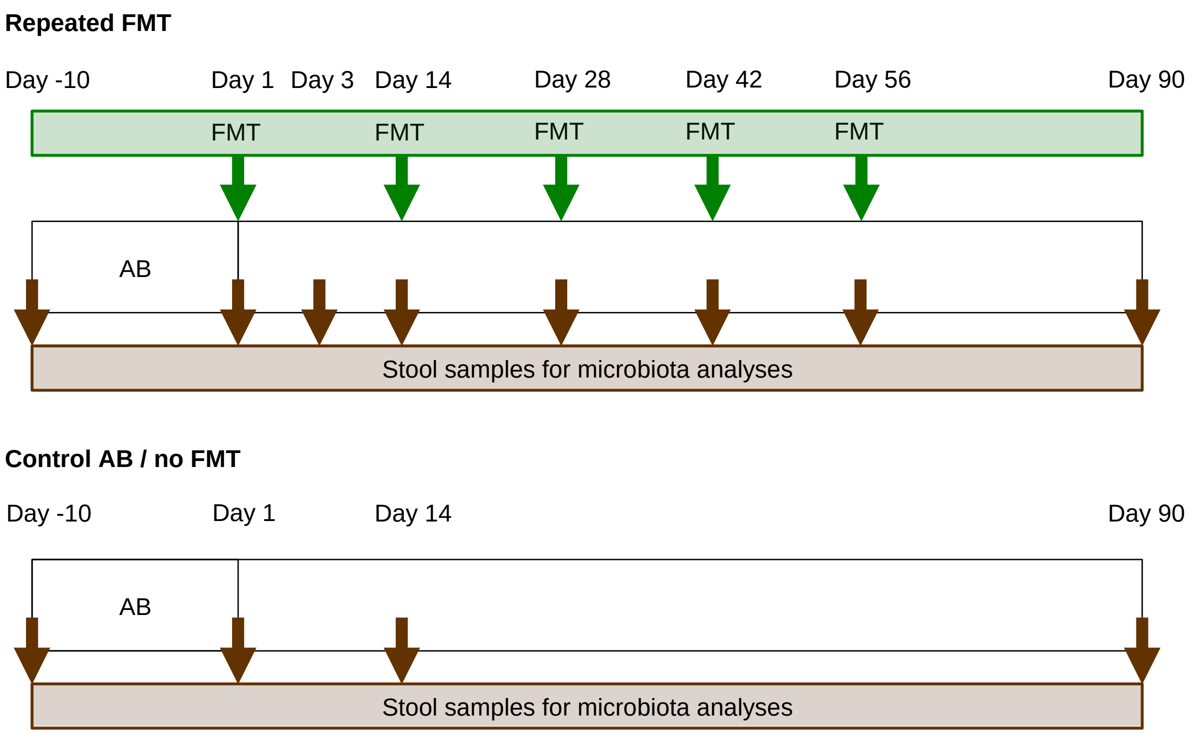

Figure S1. Study design. On the top is the scheme for the FMT-group, on the bottom for the AB-control group. Green arrows indicate when faecal microbiota transplantation was applied, brown arrows indicate when faecal samples for microbiota analysis were obtained. AB, antibiotics; FMT, faecal microbiota transplantation.

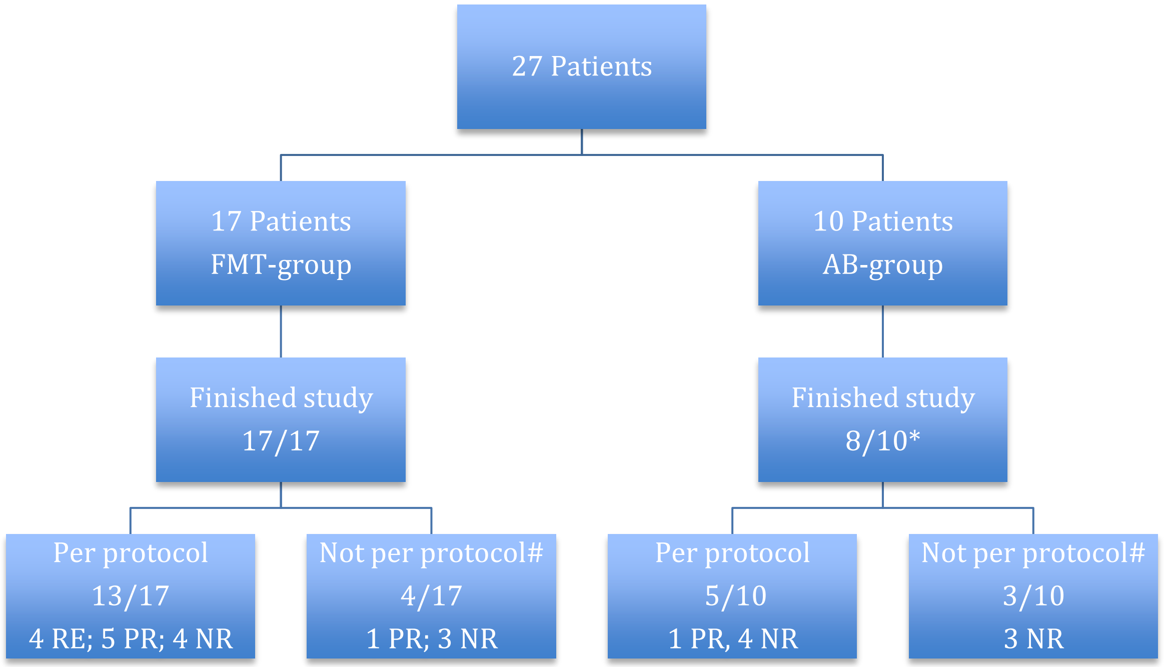

Figure S2. Number of patients in FMT and AB-control group included in study and treated per protocol.

*2 patients in the AB-group did not finish the study, one because of worsening of ulcerative colitis and one due to antibiotic associated diarrhoea (*C. difficile* negative)
3 patients in the FMT-group (1 PR, 2 NR) had prolonged faecal microbiota transplantation intervals due to donor unavailability between day 14 and day 56 respectively, 1 patient in the FMT- group started low dose steroids on his own because of increased stool frequency after day 3 and was considered as a non responder. In the AB-group 3 patients were tested positive for *C. difficile* (positive culture and positive Toxin-B gene PCR-analysis) after day 14 and received further antibiotic treatment with Vancomycin 250mg qid for 10 days. Microbiota analysis was only performed in the patients treated per protocol who finished the study.

AB, antibiotics; FMT, faecal microbiota transplantation; RE, remission; PR partial response; NR, no response

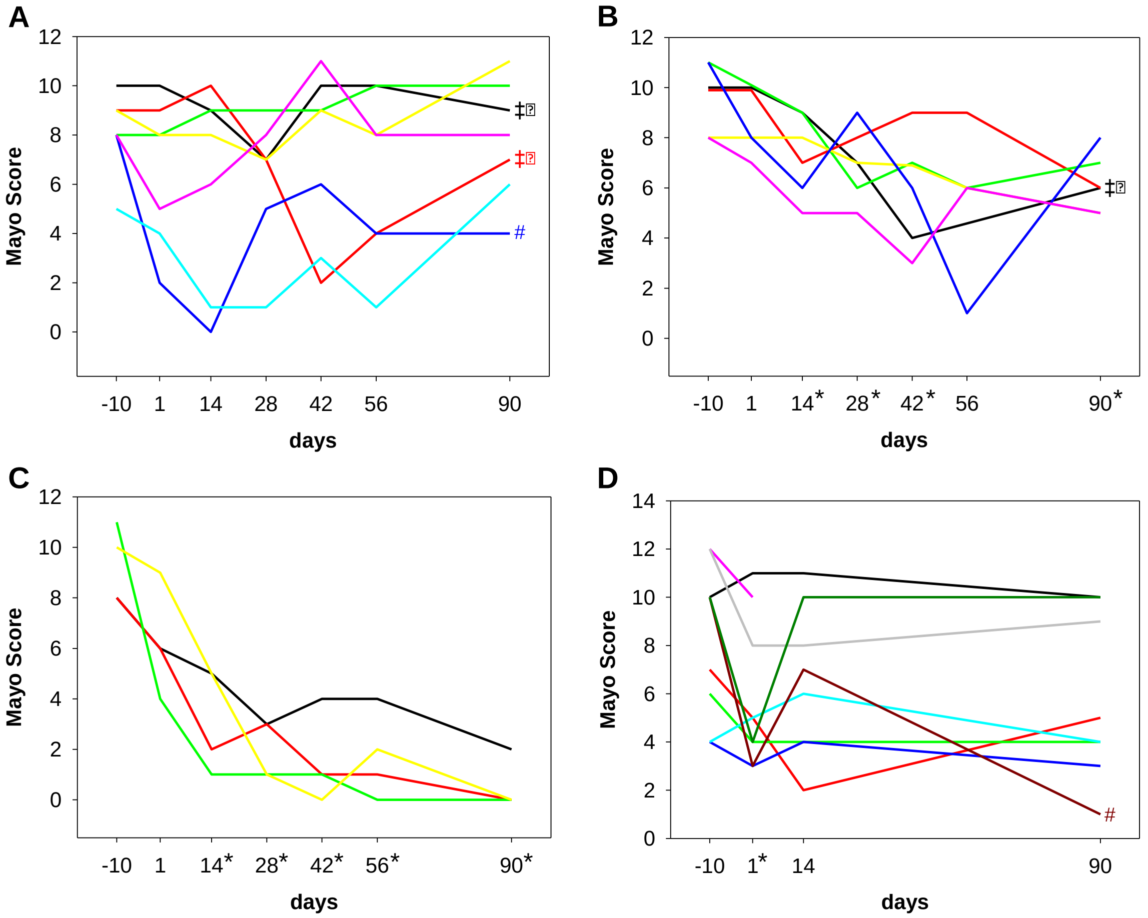

Figure S3. Time course of the total Mayo score in the individual patients. Each line denotes an individual patient during the study visits of the FMT group (A, B & C) or AB-only-group (D). A. FMT-group no response. B. FMT-group partial response. C. FMT-group remission. D. AB-group without FMT.

# indicates patients with protocol violation that were considered as non-responders, ‡ indicates patients with prolonged FMT intervals; * $p<0.05$, paired t-test, day -10 vs. other days or signed rank test when normality test failed (AB-group; day -10 vs 1). AB, antibiotics; FMT, faecal microbiota transplantation.

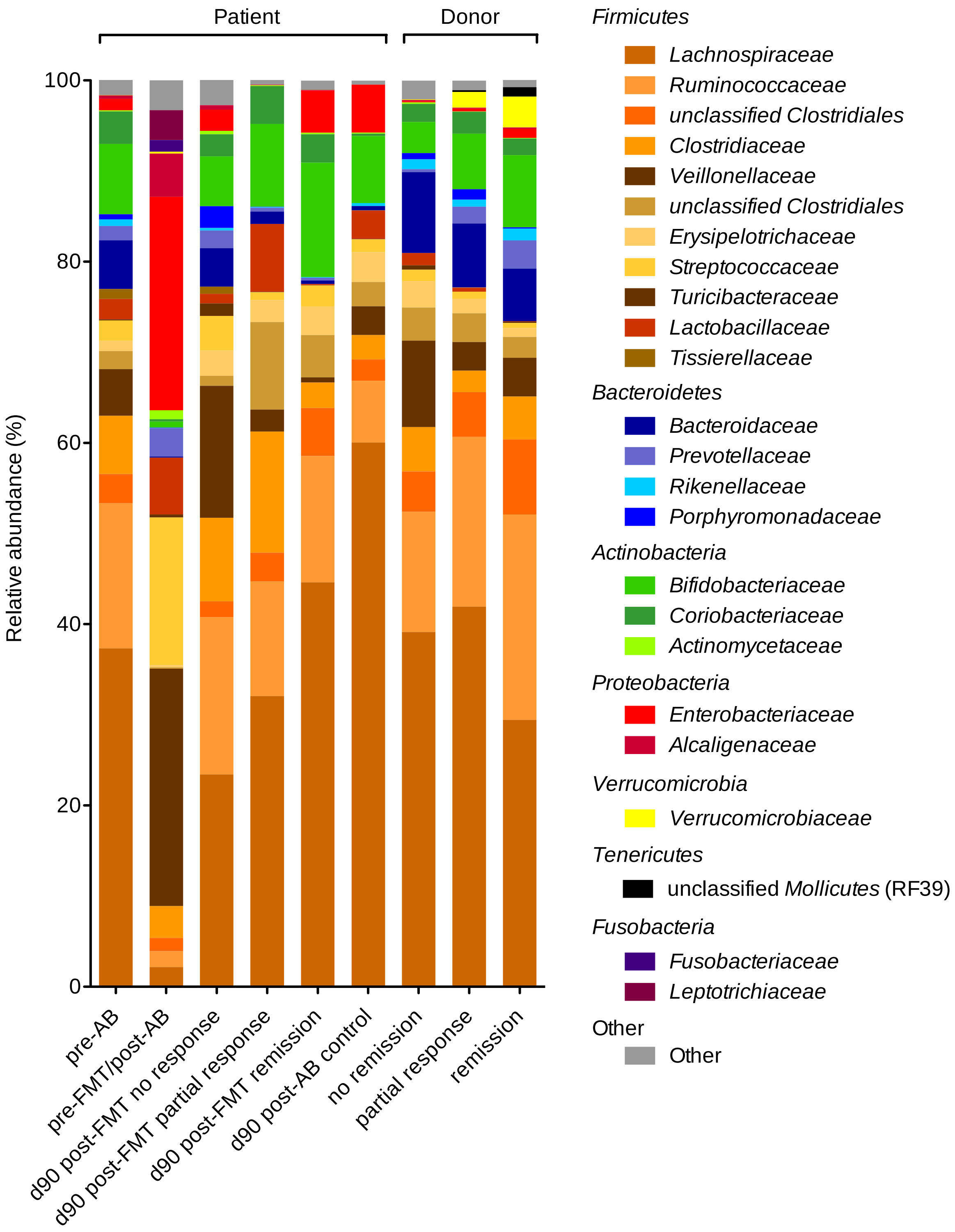

Figure S4. Taxonomic composition of the patient and donor microbiota at different time points during the study. Patient and donor microbiota composition are based on family level and are presented according to treatment response. AB, antibiotics; FMT, faecal microbiota transplantation.

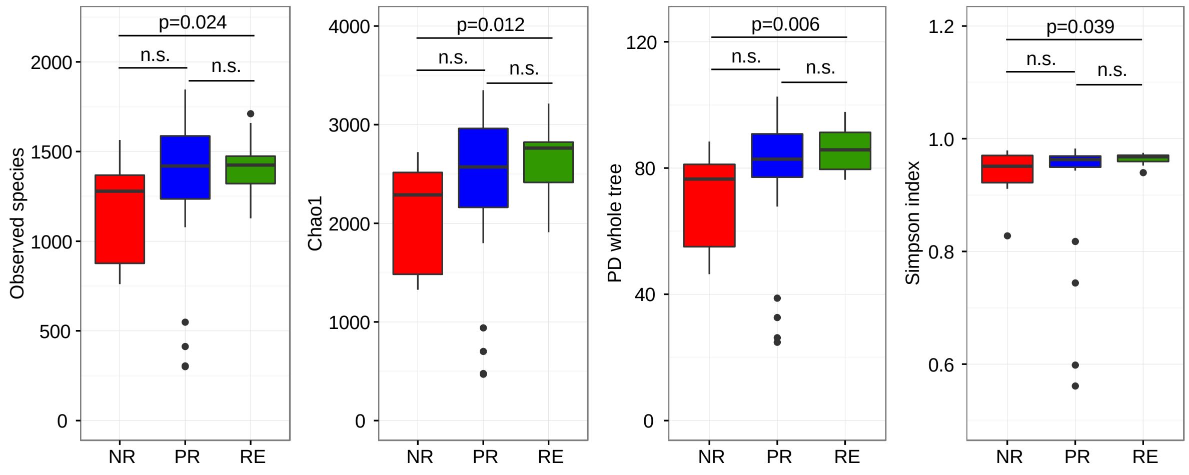

Figure S5. Microbial diversity of donor stools stratified according to patient's FMT response. A statistically significant difference was observed between donors inducing remission (RE) compared to donors inducing partial response (PR) or no response (NR). The observed species, Chao1, PD whole tree and Simpson indices are shown (red: NR; blue: PR; green: RE; n=12-23; 32672 reads/sample; nonparametric t-test, 999 Monte Carlo permutations, Bonferroni post-test).

A

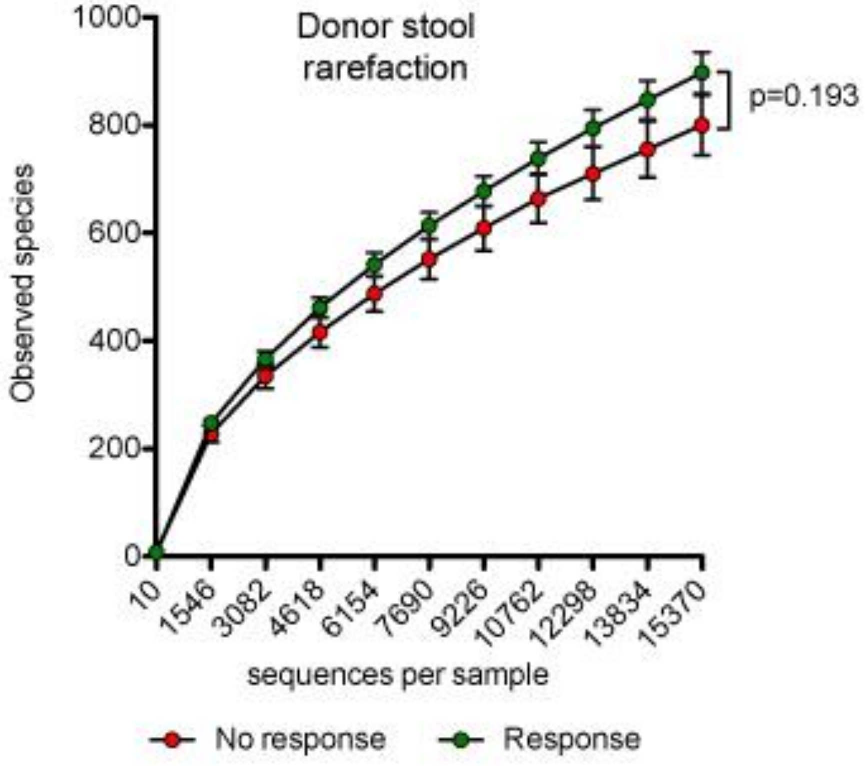

B

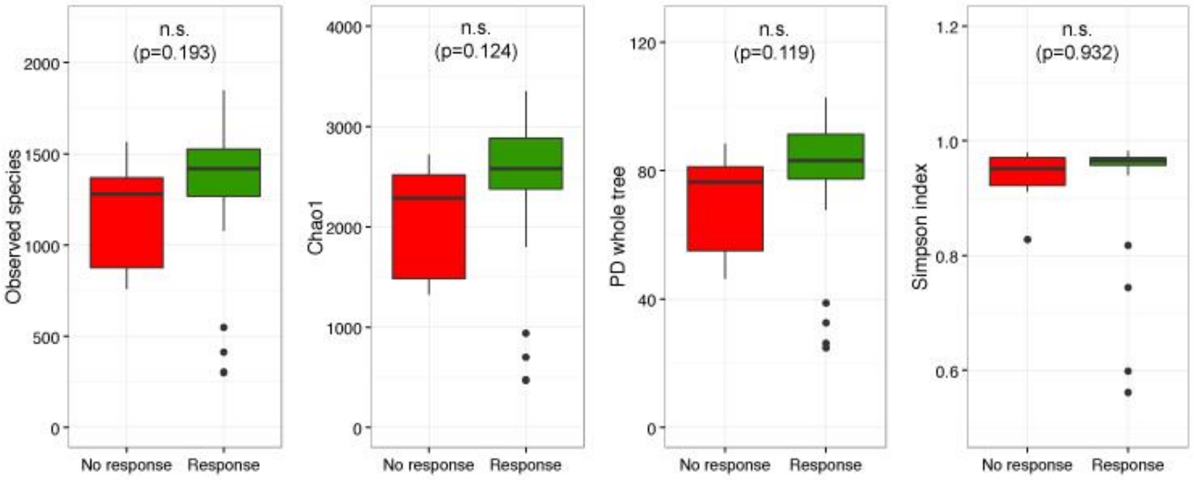

Figure S6. Alpha diversity comparing the donor microbiota leading to response and the donor microbiota leading to no response. A. Donor stools leading to response (remission + partial response) in recipients showed no difference in richness compared to donor stools leading to no response (red: no response n=12; green: response, n=39; p=0.193). Plots show mean ± SEM, p-values given for difference at 15376 sequences per sample based on two-sample t-test. B. The observed species, Chao1, PD whole tree and Simpson indices are shown (red: no response n=12; green: response, n=39; 15 376reads/sample; nonparametric t-test, 999 Monte Carlo permutations, Bonferroni post-test)

A

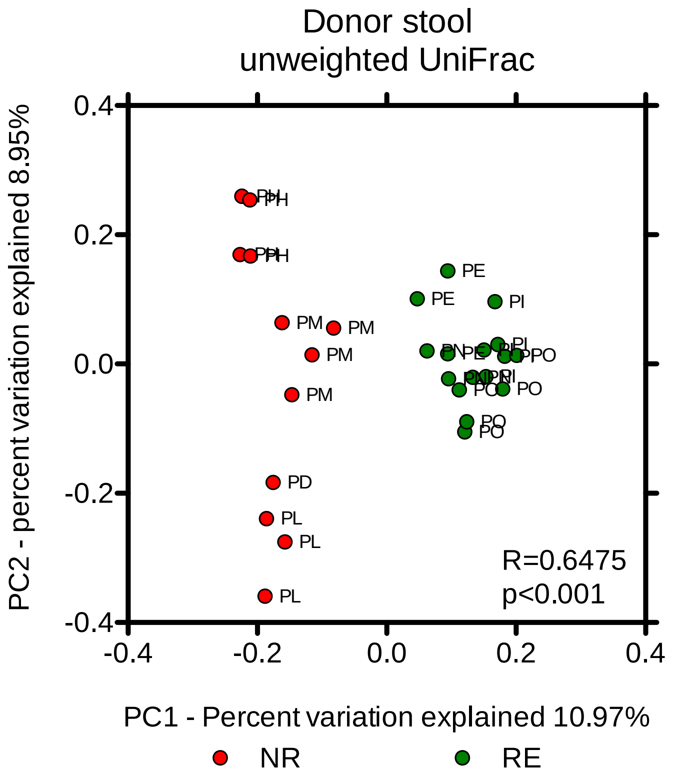

B

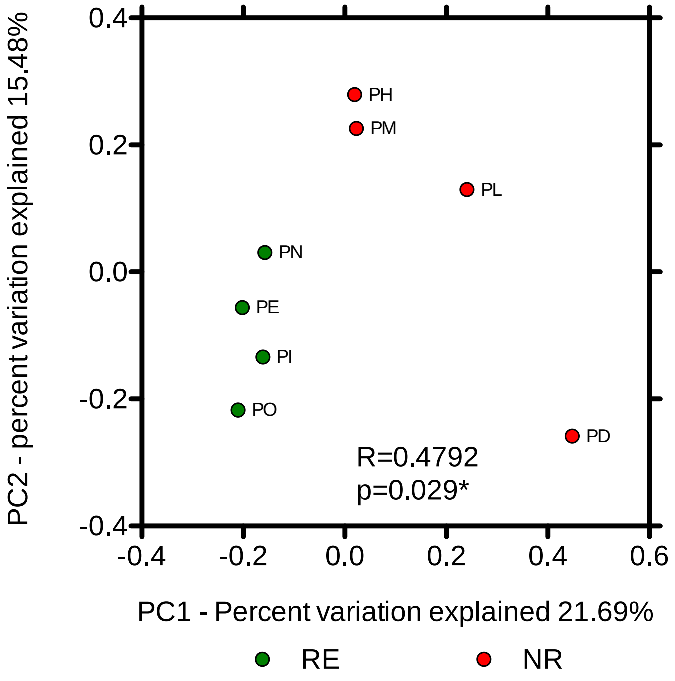

Figure S7. Donor microbiotas grouped according to faecal microbiota transplantation treatment response shown for single treatment. A Same as Figure1D labelled according to respective patient IDs with significant separation between both groups. **B** Shown is only the first donor sample available used for each patient. Dissimilarity of the donor´s microbiota analysed by unweighted UniFrac shows a significant separation. Red dots: no response, NR (n=4); green dots: remission, RE (n=4; ANOSIM).

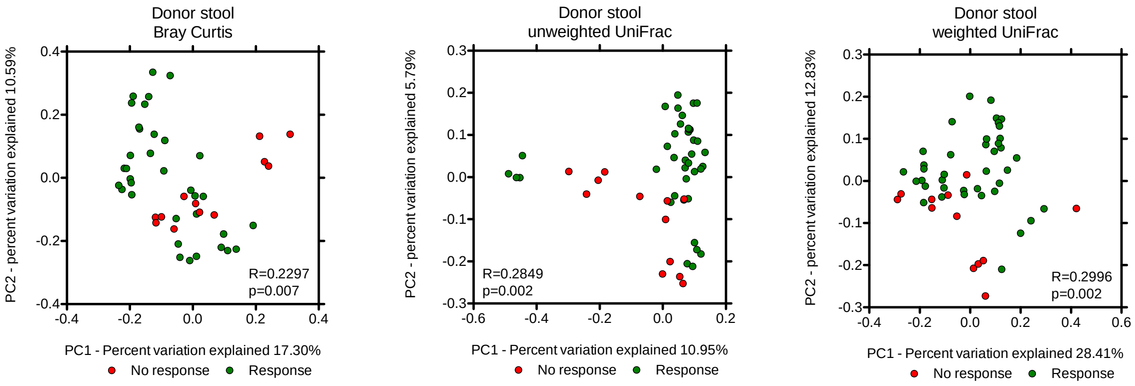

Figure S 8. Donor microbiotas grouped according to faecal microbiota transplantation treatment response. Dissimilarity of the donor´s microbiota analysed by Bray Curtis distance (left panel), unweighted UniFrac (medium panel) and weighted UniFrac (right panel) distance. Red dots: no response, n=12; green dots: response (remission + partial response), RE. (n=39; ANOSIM).

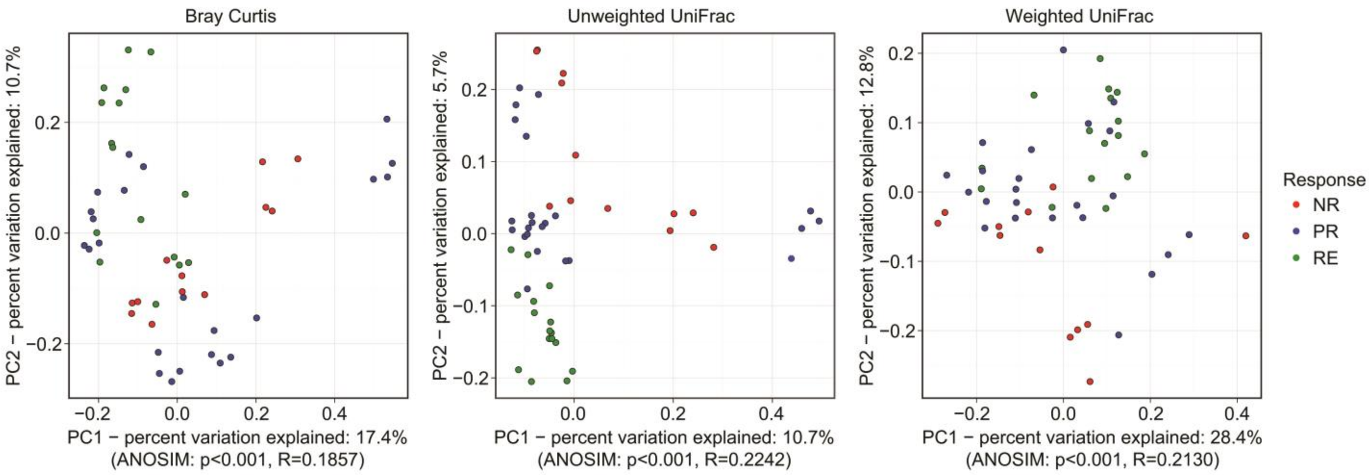

Figure S9. Donor microbiotas grouped according to faecal microbiota transplantation treatment response. Dissimilarity of the donor´s microbiota analysed by Bray Curtis distance (left panel), unweighted UniFrac (medium panel) and weighted UniFrac (right panel) distance. Red dots: no response, NR; blue dots: partial response, PR; green dots: remission, RE. (n=12-23; ANOSIM).

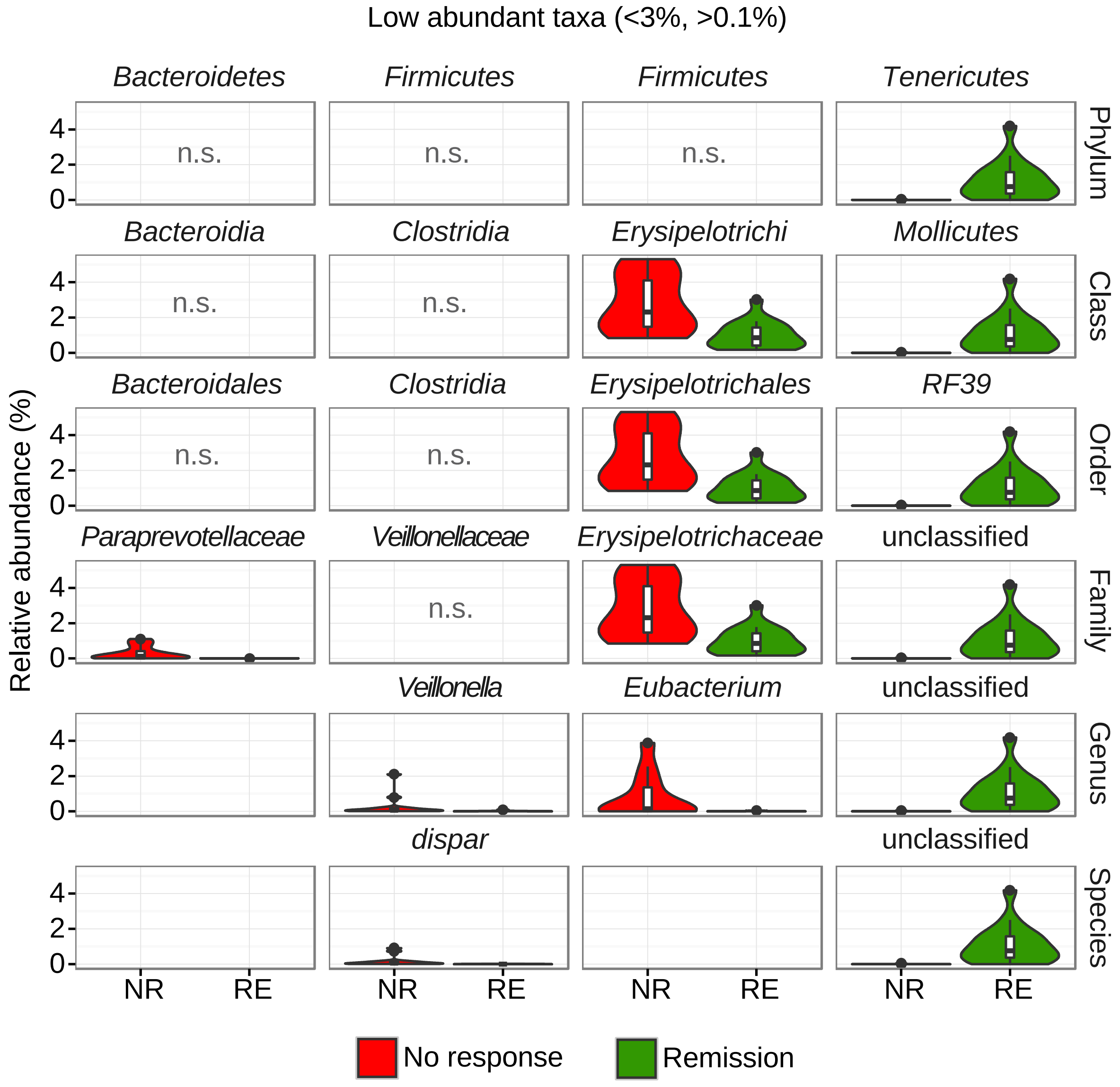

Figure S10. Low abundant taxa in donor stools associated with treatment response. Significantly different taxa from phylum to species level in the donors' microbiota associated to no response and remission in the recipient according to low abundant taxa (mean <3% and >0.1%). Plotted taxa show are significantly different between both treatment response groups based on a non- parametric Kruskal-Wallis test with FDR correction in QIIME ($p<0.05$) as well as on discriminatory features calculated by LEfSe ($p<0.05$ and LDA-score > 2; n=12-16; red: no response, NR; green: remission, RE).

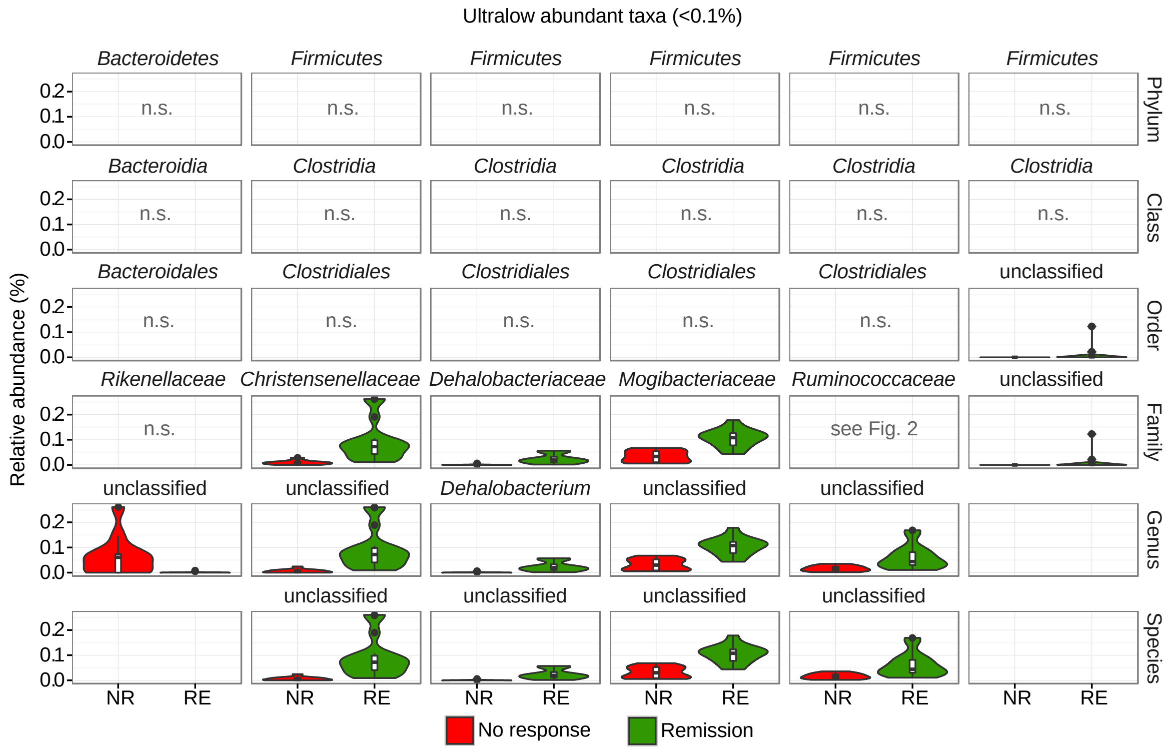

Figure S11. Ultralow abundant taxa in donor stools associated with treatment response. Significantly different taxa from phylum to species level in the donors' microbiota associated to no response and remission in the recipient according to ultralow abundant taxa (<0.1%). Plotted taxa show are significantly different between both treatment response groups based on a non- parametric Kruskal-Wallis test with FDR correction in QIIME (p<0.05) as well as on discriminatory features calculated by LEfSe (p<0.05 and LDA-score > 2; n=12-16; red: no response, NR; green: remission, RE).

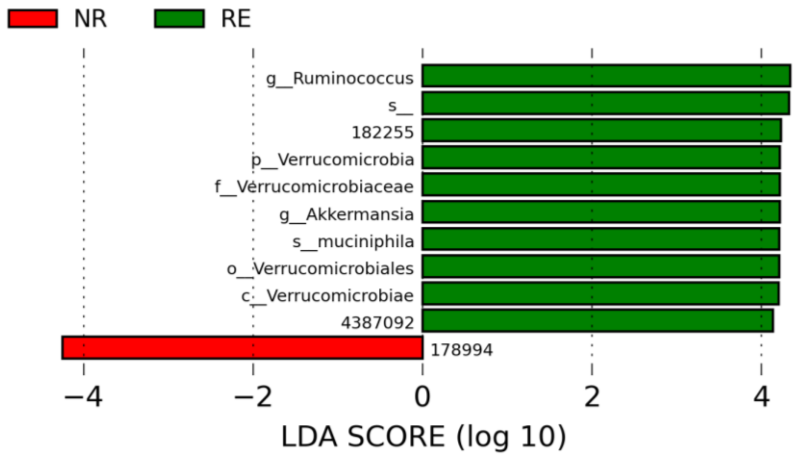

Figure S12. LEfSe analysis including only one stool sample per donor according to FMT response between donors inducing remission and donors inducing no response. Plotted taxa incl. *Akkermansia muciniphila* show are significantly different between both treatment response groups based on a non-parametric Kruskal-Wallis test with FDR correction in QIIME ($p<0.05$) as well as on discriminatory features calculated by LEfSe ($p<0.05$ and LDA-score > 4; red: no response, NR, n=4; green: remission, RE, n=4; similar samples as in Figure S7B).

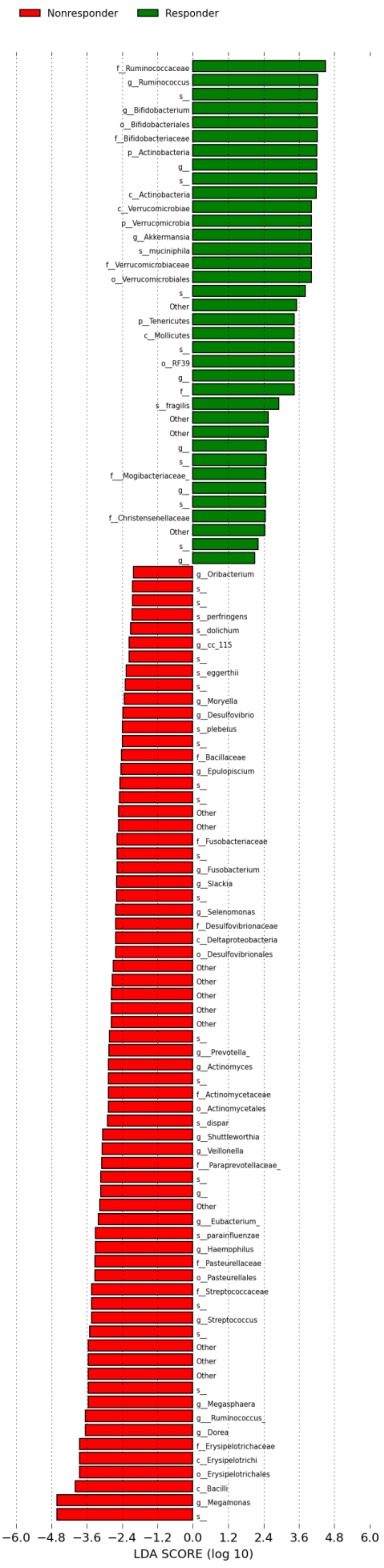
Nonresponder
Responder
f__Ruminococcaceae
g__Ruminococcus
s__
g__Bifidobacterium
o__Bifidobacteriales
f__Bifidobacteriaceae
p__Actinobacteria
g__
s__
c__Actinobacteria
c__Verrucomicrobiae
p__Verrucomicrobia
g__Akkermansia
s__muciniphila
f__Verrucomicrobiaceae
o__Verrucomicrobiales
s__
Other
p__Tenericutes
c__Mollicutes
s__
o__RF39
g__
f__
s__fragilis
Other
Other
g__
s__
f__Mogibacteriaceae_
g__
s__
f__Christensenellaceae
Other
s__
g__
g__Oribacterium
s__
s__
s__perfringens
s__dolichum
g__cc_115
s__
s__eggerthii
s__
g__Moryella
g__Desulfovibrio
s__plebeius
s__
f__Bacillaceae
g__Epulopiscium
s__
s__
Other
Other
f__Fusobacteriaceae
s__
g__Fusobacterium
g__Slackia
s__
g__Selenomonas
f__Desulfovibrionaceae
c__Deltaproteobacteria
o__Desulfovibrionales
Other
Other
Other
Other
Other
s__
g___Prevotella_
g__Actinomyces
s__
f__Actinomycetaceae
o__Actinomycetales
s__dispar
g__Shuttleworthia
g__Veillonella
f___Paraprevotellaceae_
s__
g__
Other
g___Eubacterium_
s__parainfluenzae
g__Haemophilus
f__Pasteurellaceae
o__Pasteurellales
f__Streptococcaceae
s__
g__Streptococcus
s__
Other
Other
Other
s__
g__Megasphaera
g___Ruminococcus_
g__Dorea
f__Erysipelotrichaceae
c__Erysipelotrichi
o__Erysipelotrichales
c__Bacilli
g__Megamonas
s__
−6.0 −4.8 −3.6 −2.4 −1.2 0.0 1.2 2.4 3.6 4.8 6.0
LDA SCORE (log 10)

Figure S13. LEfSe analysis of donor microbiota according to FMT response between donors inducing response and donors inducing no response. Plotted taxa are significantly different between both treatment response groups based on discriminatory features calculated by LEfSe ($p<0.05$ and LDA-score > 2; red: no response, n=12; green: response (remission + partial response; n=39).

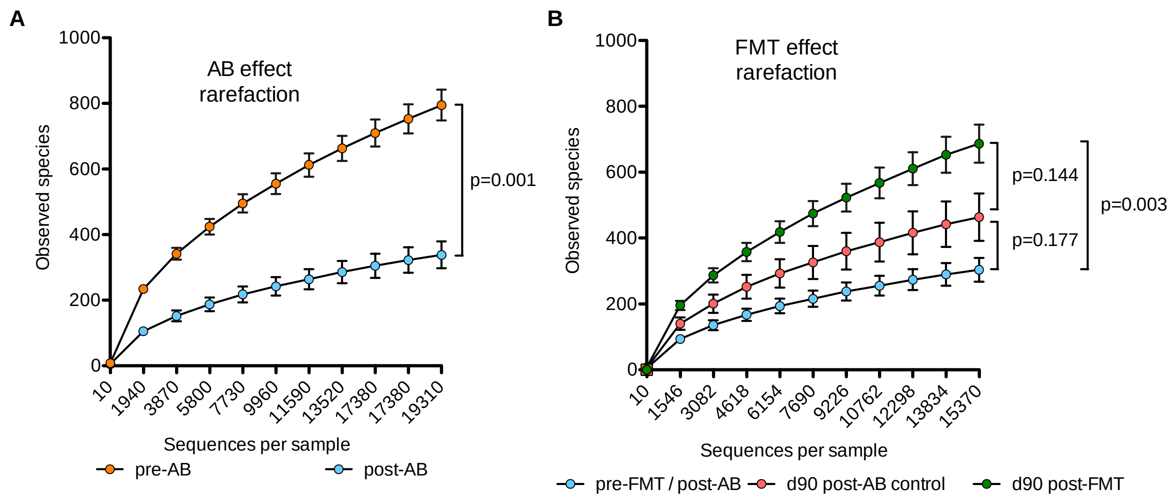

Figure S14. Influence of antibiotic pretreatment on species richness. A: Richness significantly drops after antibiotic (AB) treatment (orange: pre-AB; light-blue: post-AB; n=17-25; p-value = 0.001; mean ± SEM,19315 reads/sample; two-sample t-test.) **B:** Faecal microbiota transplantation (FMT) significantly increased species richness in contrast to controls treated with antibiotics alone on day 90 compared to day 1 (post antibiotic therapy). Alpha diversity (observed species); day 1 after antibiotic pre-treatment, day 90 antibiotic control group, day 90 FMT-group (light-blue: pre-FMT/post-AB, day 1; pink: day 90 post-AB control; green: day 90 post-FMT; n=5-25; mean ± SEM 15376 reads/ sample; two- sample t-test).

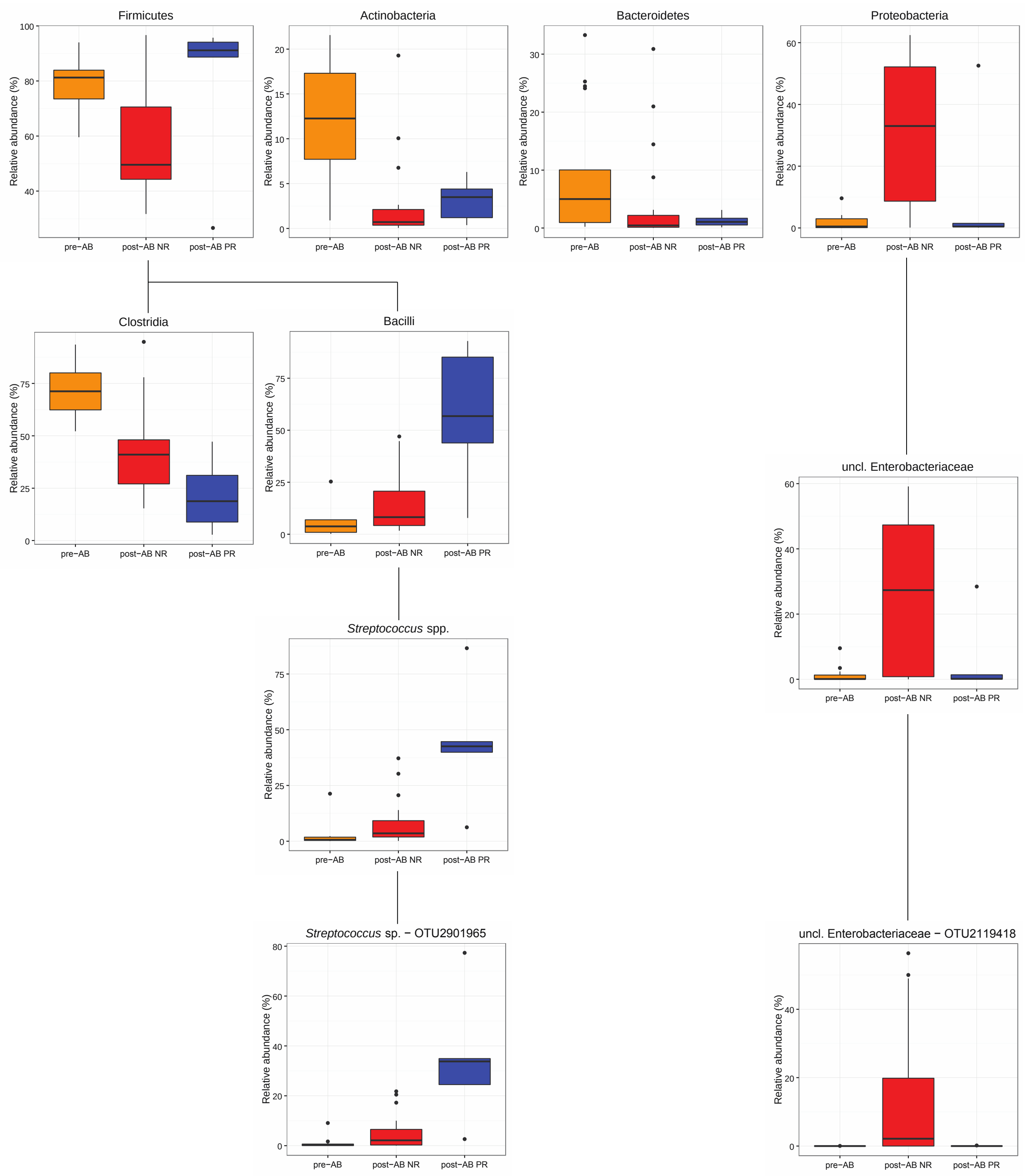

Figure S15. Faecal microbiome composition after antibiotic pretreatment according to the response of patients to antibiotics (AB). Comparing patient stool samples at day -10 pre-AB treatment and on day 1 post-AB treatment; 5 patients that showed a clinical response (partial response) according to the Mayo score and 20 patients showing no response. Stool samples pre-AB have an increased relative abundance of *Actinobacteria* and *Bacteroidetes* compared to post-AB samples. Samples post-AB correlated to a partial response show an increased abundance of *Firmicutes* and patients with no response having an increased relative abundance of *Proteobacteria*. Whereas patients pre-AB are dominated by class of *Clostridia* (*Firmicutes*) partially responding patients after antibiotics are colonized by *Bacilli* (*Firmicutes*), wherein the dominant taxon is represented by Streptococcus sp., e.g. OTU 2901965, which shows the highest similarity to *S. thermophilus* according to blast analysis. Additionally, samples of cases with no response after antibiotic treatment show increased relative abundances of *Enterobacteriaceae*, e.g. OTU

2119418, which show the highest similarity to *Klebsiella/Enterobacter* according to blast, which is barely present in responding patients. (orange: pre-AB; red: post-AB no response; blue: post-AB partial response; n=5-20; significantly different taxa are indicated with LDA- Score (>4.6), p-value (<0.05) and associated category according to LEfSe.)

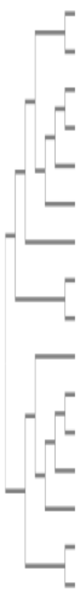

MAFFT	OTU	Genus (Qiime)	NCBI BLAST, best identity	RE (r.a.)	NR (r. a.)
	146554	*Ruminococcus*	*R. Bromii*, 99%	2.51%	0.64%
	163243	*Ruminococcus*	*R. Bromii*, 98%	0.34%	0.00%
	181961	*Ruminococcus*	*R. champanellensis*, 97%	0.41%	0.00%
	323135	*Ruminococcus*	*R. callidus*, 95%	0.36%	0.00%
	48084	*Ruminococcus*	*R. champanellensis*, 97%	0.24%	0.00%
	147969	*Ruminococcus*	*R. champanellensis*, 91%	0.40%	0.00%
	183439	unclassified	[*Clostridium*] *celleobioparum*, 93%; (*R. Albus,* 89%)	1.08%	0.02%
	363017	unclassified	[*Clostridium*] *celleobioparum*, 91%; (*R. Bromii,* 88%)	0.28%	0.01%
	358781	unclassified	*R. albus*, 90%	1.77%	0.02%
	185575	unclassified	*F. Prausnitzii*, 95%	2.04%	1.52%
	190171	unclassified	*Gemmiger formicilis*, 99%; (*F. Prausnitzii*, 93%)	1.33%	0.51%
	3236435	unclassified	*Gemmiger formicilis*, 98%; (*F. Prausnitzii*, 93%)	0.26%	0.18%
	265871	unclassified	*F. Prausnitzii*, 98%	1.15%	1.88%
	198956	*Faecalibacterium*	*F. Prausnitzii*, 98%	2.03%	1.21%
	185763	*Faecalibacterium*	*F. Prausnitzii*, 99%	0.45%	1.20%
	185390	*Faecalibacterium*	*F. Prausnitzii*, 99%	0.36%	0.23%

Figure S16. Most prevalent operational taxonomic units (OTU) sequences within family *Ruminococcaceae* of donor stool samples leading to remission. The 16 most prevalence OTUs in donor samples leading to remisson were blasted against the 16S ribosomal RNA sequences (Bacteria and Archea) database (update date: 2017/03/16; number of sequences: 18590) based on best identity. Phylogramm was created using multiple sequence alignement, MAFFT. Relative abundances of *Ruminococcus bromii* and *Ruminococcus champanellensis* OTUs show differences in donor stools leading to remission compared to donor stools with no response.This is not evident for *Faecalibacterium prausnitzii* OTUs.

Table S1. Patient characteristics according to faecal microbiota transplantation treatment response

	No Response	Response*	Remission	p value§	p value ‡
**Number of patients** n	7	10	4		
**Total Mayo score at day -10**, mean ± SD	8.1 ± 1.6	9.5 ± 1.4	9.3 ± 1.5	0.13†	0.37†
**Endoscopic Mayo subscore at day -10**, mean ± SD	2.3 ± 0.8	2.8 ± 0.4	2.5 ± 0.6	0.092	0,64
**Total Mayo score at day 1,** mean ± SD	6.6 ± 2.9	7.8 ± 2.0	6.3 ± 2.1	0.32	0.85
**Endoscopic Mayo subscore at day 1**, mean ± SD	2.0 ± 0.8	2.6 ± 0.7	2.0 ± 0.8	0.13	1.0
**Total Mayo score at day 90**, mean ± SD	8.0 ± 2.4	3.9 ± 3.1	0.5 ± 1.0	**0.013**	**0.0001**
**Endoscopic Mayo subscore at day 90**, mean ± SD	2.3 ±1.0	1.5 ± 1.3	0.3 ± 0.5	0.19	**0.004**
**Δ total Mayo score with AB treatment,** mean ± SD	1.6 ± 2.1	1.7 ± 2.0	3.0 ± 2.3	0.36	0.26
**Age**, years, mean ± SD	49 ± 22	41 ± 15	34 ± 11	0.38	0.25
**Disease duration,** years mean ± SD	9.7 ± 8.8	7.4 ± 8.1	5.8 ± 5.5	0.59†	0.44†
**Concomitant drug treatment,** n (%)					
Immunosuppressants	1/7 (14%)	4/10 (40%)	1/4 (25%)	0.78†	0.62†
Anti-TNF	0/7	1/10 (10%)	0/4	1.00†	1.00†
Systemic corticosteroids	4/7 (57%)	6/10 (60%)	2/4 (50%)	0.65†	0.65†
**Extent of disease,** n (%)					
Montreal E1	1/7 (14%)	0	0	0.34†	0.17†
Montreal E2	3/7 (43%)	7/10 (70%)	4/4 (100%)		
Montreal E3	3/7 (43%)	3/10 (30%)	0		
**Disease severity by the total Mayo score at day -10,** n (%)					
Mild (3-5 points)	1/7 (14%)	0	0	0.065†	0.54†
Moderate (6-10 points)	6/7 (86%)	7/10 (70%)	3/4 (75%)		
Severe (11-12 points)	0	3/10 (30%)	1/4 (25%)		

* including patients with partial response and remission; § between non responders and responders; ‡ between non responders and remission; Δ total Mayo score with AB treatment denotes the drop in the total Mayo score after the 10 day antibiotic pretreatment; † non-parametric test, day -10: before antibiotic treatment, day 1: before 1st faecal microbiota transplantation, day 90: end of study follow up

AB, antibiotics; SD, standard deviation; Anti-TNF, anti-tumor necrosis factor alpha antibodies

Table S2. Univariate analysis for factors predicting treatment response and remission after treatment in the whole intention to treat population (n=27)

	Response		p value
	**Yes (n=11)**	**No (n=16)**	
**Sex**, m/f	10/1	7/9	**0.018**
**Age**, years, mean ± SD	42 ± 14	40 ± 18	0.78
**Extent of disease,** Montreal classification, E1/E2/E3	0/8/3	2/9/5	0.43
**Duration of disease**, years, median (range)	5.0 (1-28)	4.5 (1-22)	0.44
**Mayo Score before treatment**, median (range)	10 (8-12)	8 (4-12)	**0.037**
**Endoscopic Mayo subscore before treatment**, median (range)	3 (2-3)	2.5 (1-3)	0.082
**Prior anti-TNF**, y/n	7/4	7/9	0.44
**Prior immunosuppressants**, y/n	7/4	13/3	0.39
**Ongoing anti-TNF**, y/n	2/9	1/15	0.55
**Ongoing immunosuppressants**, y/n	5/6	6/10	0.71
**Ongoing corticosteroids**, y/n	5/6	8/8	1.0
**Ongoing Mesalazine**, y/n	9/2	8/8	0.12
**FMT**, y/n	10/1	7/9	**0.018**
	**Remission**		
	**Yes (n=4)**	**No (n=23)**	
**Sex**, m/f	3/1	14/9	0.52
**Age**, years, mean ± SD	34 ± 11	42 ± 17	0.37
**Extent of disease**, Montreal classification, E1/E2/E3	0/4/0	2/13/8	0.25
**Duration of disease, years**, median, (range)	4.5 (1-13)	5 (1-28)	0.58
**Mayo Score before treatment**, median, (range)	9 (8-11)	9 (4-12)	0.63
**Endoscopic Mayo subscore before treatment**, median (range)	2.5 (2-3)	3 (1-3)	0.69
**Prior anti-TNF**, y/n	2/2	12/11	0.67
**Prior immunosuppressants**, y/n	3/1	17/6	1.0
**Ongoing anti-TNF**, y/n	0/4	3/20	1.0
**Ongoing immunosuppressants**, y/n	1/3	10/13	0.62
**Ongoing corticosteroids**, y/n	1/3	12/11	0.60
**Ongoing Mesalazine**, y/n	2/2	15/8	0.61
**FMT**, y/n	4/0	13/10	0.26

FMT, faecal microbiota transplantation; anti-TNF, anti-tumor necrosis factor alpha antibodies; SD, standard deviation

Table S3. Laboratory parameters in patients according to faecal microbiota transplantation treatment response.

	No Response	Response*	Remission	p value§	p value‡
**Number of patients** (n)	**7**	**10**	**4**		
**Laboratory values at day -10**					
WBC (×10^9/L)	11.9 ± 3.0	8.4 ± 3.2	7.1 ± 1.9	0.057	**0.023**
Hemoglobin g/dL	13.6 ± 1.8	13.8 ± 1.1	13.9 ± 1.8	0.86	0.81
Thrombocytes (×10^9/L)	361 ± 82	297 ± 94	277 ± 126	0.19	0.23
CRP mg/L	7.0 ± 4.3	9.5 ± 12.0	3.6 ± 4.2	0.96†	0.29†
Fecal calprotectin, µg/g	380 ± 127	1317 ± 469	1244 ± 342	**0.013**	0.15
**Laboratory values at day 1**					
WBC (×10^9/L)	9.8 ± 2.7	7.0 ± 2.5	5.1 ±1.0	**0.044**	**0.003**
Hemoglobin g/dL	13.6 ± 2.3	13.2 ± 1.3	13.0 ± 2.1	0.68	0.69
Thrombocytes (×10^9/L)	341 ± 86	252 ± 59	260 ± 91	**0.023**	0.17
CRP mg/L	13.5 ± 15.0	4.1 ± 5.2	2.0 ± 2.1	0.27†	0.18†
Fecal calprotectin, µg/g	406 ± 258	664 ± 603	254 ± 270	0.34	0.40
**Laboratory values at day 90**					
WBC (×10^9/L)	8.6 ± 3.7	6.6 ± 1.9	5.4 ± 1.6	0.26	0.15
Hemoglobin g/dL	13.5 ± 1.8	13.3 ± 1.3	13.3 ± 2.0	0.73	0.83
Thrombocytes (×10^9/L)	352 ± 110	276 ± 58	280 ± 73	0.09	0.29
CRP mg/L	21.7 ± 20.4	4.1 ± 6.3	1.8 ± 1.3	**0.022†**	**0.033†**
Fecal calprotectin, µg/g	867 ± 593	552 ± 622	309 ± 394	0.34	0.14

all values are means ± SD; * including patients with partial response and remission; § between non responders and responders; **‡** between non responders and remission; p-values were obtained by unpaired t-test, p-values indicated by † were obtained by Mann Whitney-U-test; day -10: before antibiotic treatment, day 1: before 1st faecal microbiota transplantation, day 90: end of study follow up SD, standard deviation; WBC, white blood cells; CRP, C-reactive protein
